# Translational Models of Arrhythmia Mechanisms and Susceptibility: Success and Challenges of Modeling Human Disease

**DOI:** 10.3389/fcvm.2019.00135

**Published:** 2019-09-10

**Authors:** Joseph S. Piktel, Lance D. Wilson

**Affiliations:** Department of Emergency Medicine, Emergency Care Research Institute and Heart and Vascular Research Center, MetroHealth Campus of Case Western Reserve University, Cleveland, OH, United States

**Keywords:** animal models, translational models, sudden cardiac death, resuscitation, arrhythmias

## Abstract

We discuss large animal translational models of arrhythmia susceptibility and sudden cardiac death, focusing on important considerations when interpreting the data derived before applying them to human trials. The utility of large animal models of arrhythmia and the pros and cons of specific translational large animals used will be discussed, including the necessary tradeoffs between models designed to derive mechanisms vs. those to test therapies. Recent technical advancements which can be applied to large animal models of arrhythmias to better elucidate mechanistic insights will be introduced. Finally, some specific examples of past successes and challenges in translating the results of large animal models of arrhythmias to clinical trials and practice will be examined, and common themes regarding the success and failure of translating studies to therapy in man will be discussed.

## Introduction

Improved approaches to prevent and treat arrhythmia are of major importance, as sudden cardiac death (SCD) is a major cause of mortality (1, 2). In 2014 in the United States, over 600,000 deaths were caused by heart disease, with half of those being sudden. Ventricular fibrillation/tachycardia (VF/VT) secondary to acute myocardial ischemia or infarction (MI) is devastating, with many patients not reaching the hospital and most of those who do suffering poor outcomes (3). Therefore, improved understanding of both mechanism and novel therapies for arrhythmias derived from translational models, which can be readily applied to humans, is of utmost importance.

There are multiple large animal models used to assess arrhythmia susceptibility, mechanisms of arrhythmogenesis, and therapies for arrhythmia and SCD. In this review we will focus on large animal models designed to derive mechanisms for arrhythmias and SCD as well to test therapies in translational models to provide evidence of efficacy and the bases for clinical trials. We will discuss the pros and cons of animal models using the primary large animal platforms in arrhythmia and SCD research: dog, pig, goat, and sheep. Although smaller animal models of arrhythmia susceptibility such as rabbit models of heart failure (HF) (4) and murine models of ischemia/reperfusion (5) have contributed significantly to our knowledge of arrhythmia mechanisms, this review will focus on large vertebrate models most readily translatable to human therapies.

### Why Use Large Animal Models and What Is Their Role?

**Figure 1:**
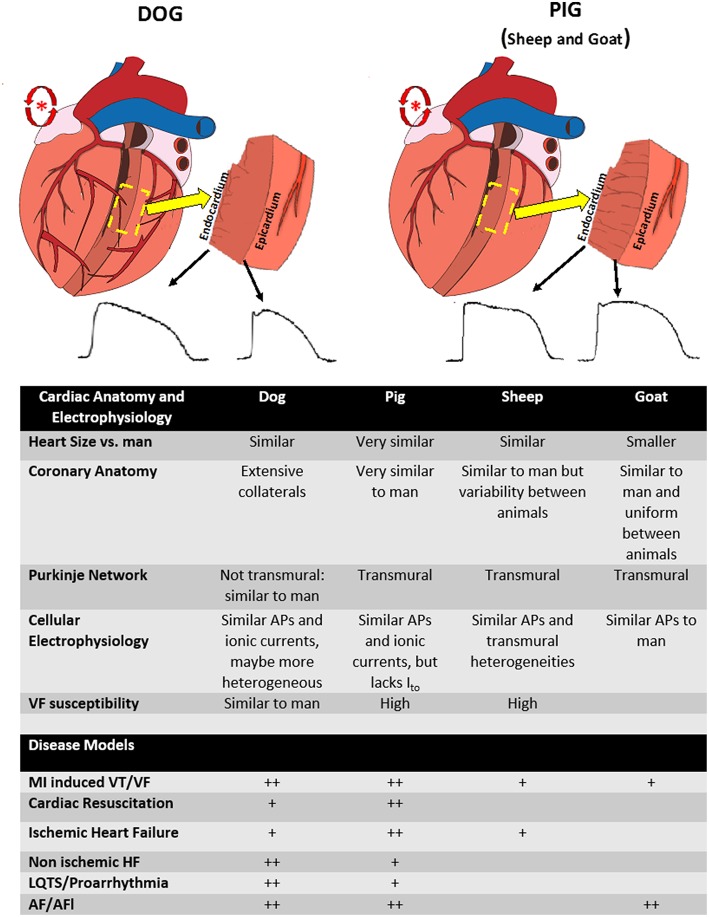
Differences between anatomy and electrophysiology between species. (Top left) Figure of the anatomy of the dog heart. Note the extensive coronary artery collaterals (red vessels), endocardially distributed purkinje network (gray lines on transmural section), and relative transmural heterogeneity in action potentials. (Top right) Figure of the anatomy of the pig heart. Note the lack of coronary collaterals, transmural purkinje system (gray lines on transmural section), and decreased transmural heterogeneity. Both species can be used to model atrial fibrillation (red atrial trigger/reentry schematic). (Lower Table) Summary of the anatomical and electrophysiological differences between animal species as well as the relative common diseases modeled in each species (+, modeled, ++, preferred model).

Large animal models are often used to determine the relevance of previously derived mechanisms in *ex vivo* or in small animal models. This is an important step in understanding how molecular and cellular mechanisms contribute to arrhythmias in man and oftentimes crucial, as the relevance of mechanisms derived *ex vivo* or in smaller animal models possessing cardiac electrophysiology and anatomy that has significant differences from man is uncertain. More typically, translational models are performed to justify therapies prior to clinical trials evaluating treatments, i.e., device or drug. Large animal models lend themselves to drug development questions, such as delivery, pharmacodynamics and pharmacokinetics studies and assessment of immune response and toxicity. However, even this remains controversial (6). Rarely are they used to guide therapy in man, with notable exceptions (epinephrine for cardiac arrest was tested in dogs and translated directly to man) (7) or in some instances of rare diseases when clinical trials are unrealistic (typically in toxicology) (8). Although no large animal model encompasses all the anatomical and electrophysiological features of man, many similarities exist. However, important differences need to be understood when attempting to translate findings in large animals to humans (9) (see Figure 1).

### Testing Arrhythmia Mechanisms and Developing Interventions Using Interventional Electrophysiology and Imaging Techniques

A specific benefit in using large animal models to study mechanisms of arrhythmia and sudden death is that they are very well-suited for assessments of arrhythmia substrates and mechanisms which can also be done in man. Given the size of the adult animal and heart, dogs, pigs, goats, and sheep all can be instrumented with standard human electrophysiology interventions, as well as testing novel catheter based therapies using standard and investigational interventional electrophysiological techniques (9–11). Many of these methods are used clinically in everyday use, or can be adapted to study mechanism. These include 3-D anatomical mapping, contact and non-contact electroanatomical mapping and ablation techniques (10, 12, 13). Imaging modalities (MRI, CT, Echocardiography, etc.) have all been used to develop methods of high resolution combined anatomic and electrical mapping and produce functional measurements of ventricular remodeling (ex. myocardial strain measurements) to determine mechansims of electrophysiological remodeling in disease (14–17).

### Which Diseases Have Been Modeled to Determine Arrhythmia Mechanisms and Susceptibility?

Translational models of arrhythmias have naturally focused on modeling diseases which are highly associated with SCD. Models of acute ischemia/reperfusion and MI (3) have been adapted to more chronic models of ischemic cardiomyopathy, with and without acute ischemia (18, 19). Important arrhythmogenic conditions closely related to MI include models of resuscitation from SCD secondary to ischemia and exercise induced VT/VF (20–22). As many as 50% of patients with HF die of arrhythmogenic SCD, so significant effort has been made to model human HF and determine arrhythmia mechanisms in other structural heart diseases, such as isolated hypertrophy (23). Models used to determine arrhythmia specific arrhythmogenic diseases predisposing to SCD have also been well-established. For example, there are specific large animal models of arrhythmogenic right ventricular cardiomyopathy (ARVC) and Duchenne muscular dystrophy (DMD) (24, 25). Large animal models of channelopathies and the drug-induced long QT syndrome (LQTS) have been developed (23, 26, 27). Finally, although not models of SCD, there are multiple, well-characterized large animal models of atrial fibrillation and flutter (AF/AFl), the most common arrhythmia in man (28, 29). Table 1 reviews some of the important specific model platforms used to derive mechanism and test therapies in selected arrhythmogenic diseases.

**Table 1:** Examples of specific models platforms used to derive mechanism and test therapies in selected arrhythmogenic diseases.

**Disease**	**Species**	**Model**	**Advantages**	**Disadvantages**	**Contributions**	**References**
Heart failure	Pig	Atrial pacing induced	Model of tachycardia induced cardiomyopathy Repolarization abnormalities and EP remodeling characterized Develop spontaneous arrhythmias	Less arrhythmia characterization than dog Unlike dog, does not have element of ventricular remodeling due to abnormal ventricular activation	Enhanced understanding of electrophysiological mechanisms of arrhythmia in HF	(30, 31)
		Ischemic (coronary artery occlusion, embolization, ligation)	More generalizable to many forms of human HF Coronary anatomy similar to human Multiple methods can be used to induce Recent in depth electrophysiological characterization	*In vivo* arrhythmia mechanisms may be less well-characterized than *ex vivo*	Enhanced understanding of electrophysiological mechanisms of arrhythmia in HF Defines importance of infarct border zone and HF development	(32, 33)
	Dog	Pacing induced	Reducible and titratable ion channel and Ca2+ handling remodeling comparable to human HF Neurohormonal changes similar to man Spontaneous arrhythmias	LV function recovers quickly after pacing stopped Not generalizable to many forms of human HF	Can be used as a model platform in drug development Defined multiple arrhythmia mechanisms in HF, including PMVT	(34, 35)
	Dog	Ischemia	Generalizable to many forms of human HF Neurohormonal changes similar to man Likely spontaneous arrhythmias	Coronary artery ligation method potentially problematic in creating uniform HF Coronary microembolism provides more uniform ischemic insult, but technically difficult		(36–38)
	Sheep	Ischemic or pacing	Similar coronary anatomy to man with uniform infarctions Electrical and mechanical remodeling similar to human HF More generalizable to many forms of human HF	Requires significant expertise	Defines importance of infarct border zone and HF development	(39–41)
Acute ischemia/ reperfusion and MI	Pig	Coronary occlusion	Similar coronary anatomy to man with uniform infarctions and area at risk Anatomy and electrophysiological response to injury reflects human Course of arrhythmia occurrence during acute ischemia is well-defined		Defined time dependence of ischemia induced arrhythmias and their mechanisms Studies have provided framework for understanding clinical approach to ischemia induced arrhythmia	(11, 42, 43)
Established infarction	Pig		Model with high fidelity to human post-MI VT Large body of experience	Requires significant expertise	More recently demonstrates the potential of gene therapy to treat reentrant arrhythmias post-MI	(44–46)
	Dog		High fidelity to human post-MI VT Large body of experience supporting mechanisms of arrhythmia, including role of autonomic NS	More established collateral flow, with infarct patterns less uniform	Defined patterns of tissue necrosis and initial time course of arrhythmia progression during ischemia and MI Defines importance of BZ in arrhythmia mechanisms and evolution of arrhythmia substrates post-infarction	(20, 38, 47)
	Sheep	Recent MI and brady cardia	Spontaneous arrhythmias Allows assessment of substrates with intact autonomic ns	Requires significant expertise Original report utilized ICD Dependent on Bradycardia after MI, which likely induces additional remodeling, so complex remodeling needs to be characterized	Given heterogeneity between subjects in VT susceptibility, model platform for interventions and mechanisms of SCD	(48)
Resuscitation	Pig	Electrically induced	Potential for uniform downtime and post-resuscitation cardiac function Established model for quality human CPR	Less translatable to the most common form of VF, ischemia-induced	Have established initial methods of quality CPR performance later validated in clinical trials	(49–54)
		Ischemic	Coronary anatomy similar to human, able to reasonably replicate size of infarct Able to identify more common arrhythmia mechanisms during myocardial ischemia Mimics most likely scenario of SCD	Increased likelihood of cardiogenic shock makes model more challenging More heterogeneities in ROSC times More heterogeneities in ACLS pharmacology	Effects of TTM on arrhythmia substrates	(22)
		Hypoxic	Most common cause of arrest in children	Less commonly associated with arrhythmias or cardiac cause of arrest		(55)
	Dog	Healed MI followed by arrest	characterization of ion channels more similar to human physiology after arrest	Coronary anatomy less well-suited for acute MI followed by VF arrest	Significant contributions to cardiocerebral resuscitation	(56, 57)
Atrial fibrillation	Pig	Atrial Pacing-induced with or without ventricular pacing	If no AV block, causes progressive ventricular tachypacing and remodeling so relevant to HF in AF in man (but not other types of AF) Sustained AF	Need to consider and/or control for effects of rapid atrial pacing on ventricular rate and remodeling	Has been used to illustrate mechanisms, including importance of ROS Model platform for gene therapy strategies to treat AF, demonstrating feasibility of this approach	(28, 58–61)
	Dog	Atrial Pacing-induced	Extremely well-characterized ionic and molecular mechanisms in the model	EP remodeling reverses after stopping pacing	Defined models of AF relevant to paroxysmal AF/AT	(28, 29, 62)
		Mixed AF and HF	Demonstrated differences in mechanism in AF related to atrial tachyarrhythmia vs. in HF Strongly integrated molecular, ionic, and structural mechanisms with arrhythmia mechanisms (all models)	Method of HF induction may not be relevant to other causes of HF in man	Defined structural basis and ionic remodeling for AF in setting of HF, and underlying mechanisms producing the structural remodeling	(28, 29, 62, 63)
		Sterile pericarditis	Model of sustained atrial flutter EP mechanisms established in the model Relatively easy to create, reproducible	Most relevant to post-operative AF/AFl and inflammation over other causes of AF	Mechanisms of post-operative AF and importance of inflammation in AF	(64)
	Goat	Atrial Pacing induced	Well-established conical model of AF Used to test efficacy of antiarrhythmic drugs Provides insight into atrial contractile dysfunction with more prolonged AF Validated models with AF associated with cardiac hypertrophy and atrial dilation	AF and EP remodeling reverts after cessation of pacing	Model of paroxysmal AF Concept that AF begets AF, and demonstrated underlying electrical remodeling responsible	(28, 65–68)
LQTS (inherited)	Pig	Brugada mutation	Exhibits many features of Brugada phenotype in man and reproduces a known Brugada mutation in large animal model	Single Brugada mutation Characterized in young pigs Elements of phenotype have yet to be characterized	First large animal translation model of heritable channelopathy Potential strong model platform for further investigations	(69, 70)
LQTS (acquired/drug-induced)	Dog	AV block dog	Very well-characterized and validated, arrhythmia mechanisms largely explored, ventricular remodeling Assessments can be done in conscious dogs	Expensive Low throughput for preclinical proarrhythmia testing Similar model has been proposed in pig	Clear utility as a screening model in drug development Has provided significant mechanistic information, including demonstration and quantification of repolarization reserve	(23, 26, 71, 72)
Disease specific AVRC	Boxer dog	Chromosome 17 variant	Very well-characterized model Spontaneous ventricular arrhythmias and sudden death Shares many features with human disease	Specific mutation	Has demonstrated importance of intercalated disc and connexin remodeling in pathogenesis of arrhythmias	(25, 73)
Duchenne's muscular dystrophy	Golden retriever dogs	X-linked dystrophin gene mutation	Develop progressive cardiomyopathy very similar cardiac phenotype as in man Very well-characterized model overall Develop spontaneous ventricular arrhythmias	Severe phenotype Other DMD mutations available in canine models Ventricular Arrhythmia phenotype and mechanisms less well-characterized	Translational model platform for multiple pharmacologically, cell therapy and genetic based studies	(24, 74–76)

## Models of Specific Arrhythmogenic Disease

### What Are the Pros and Cons of Specific Species in Modeling Human Arrhythmias?

#### Pig

Given their anatomic and physiological (heart size, coronary anatomy, inflammatory response) and electrophysiological similarities to man, pig models are arguably the preferred species for translational work and have been used to model most arrhythmogenic disease (9). There are some specific electrophysiological differences (most notably a transmural Purkinje system not present in canine or man and diminished transient outward potassium current) which need to be considered, particularly regarding models of acute ischemia (81). In addition, pigs are more susceptible to developing VF than man, which is a consideration when translating outcomes of sudden death to humans (11). Despite this, pig models have been a benchmark for acute MI, acute ischemia and reperfusion and resuscitation studies. This is likely because pigs have more anatomically similar coronary vascular anatomy to man and do not have the extensive collaterals as occurs in canine, making a transmural infarct more similar to man (11, 82, 83). There are established models of ischemic and non-ischemic HF in pigs (Figure 1). A specific consideration in pigs is that although there are established models of atrial tachyarrhythmias, with similarities to the Allessie goat model of atrial fibrillation (65) (discussed below), pigs will develop a tachycardia-induced HF due to the intrinsically fast AV conduction, which needs to be taken into account when designing models of AF (84). Advantages include cost and availability of pigs for research, ability to induce coronary artery disease (85), and that they are amenable to long term outcome studies. Because of their anatomic similarities, well-validated ability to perform cardiopulmonary resuscitation, including CPR, pigs have become the standard translational model for most aspects of human resuscitation, including studies of CPR methodology, arrhythmia mechanisms, and pharmacological treatment (51, 52, 55, 86). Most commonly, VF is induced by electrical current, either on the epicardial surface in an open chest model, or through an intracardiac catheter in a closed chest model. Large animal models also allow for translationally relevant models of myocardial ischemia induced VF and cardiac arrest, due to the ability to perform a transmural infarct (55). As the etiologies of cardiac arrest are also multifaceted, several resuscitation models have been developed, providing important insights (see Table 1).

#### Canine

Historically, canine models have been used to evaluate cardiac arrhythmia and remain and important model platform. They have similar cellular electrophysiology to man, with similar cellular ionic currents governing depolarization and repolarization and therefore similar action potential profiles. Although controversial, the transmural action potential gradients, producing transmural dispersion of repolarization may be more similar to man (87–89). This makes them perhaps more amenable to studies of acquired or drug-induced long QT syndrome, although this is controversial (23, 27). Similar to man, they do not have a transmural Purkinje system (9, 90). There are other specific advantages to canine models, including better understood autonomic control, which may be more pronounced (slower baseline HR, larger range of HR) (91). They also may be more appropriate for evaluation of autonomic modulation of arrhythmias given their social nature and that they can be trained for conscious experiments which may be particularly important when confounding factor of anesthesia is critical, such as in neural modulation of arrhythmias (20). As in man, dogs can develop valvular disease with aging (92). Much of what we know about mechanisms of arrhythmia in ischemia/reperfusion have also been derived from dog models. There are also a variety of canine HF models (19, 35). A number of AF models in dog have been developed, but AF is not sustained in most dog models (28).

#### Sheep

Advantages to sheep models include structural heart anatomy that is very similar to man, including similar coronary anatomy and ventricular remodeling after MI (9, 93). However, there has been reported to be significant variability in coronary anatomy between subjects, although uniform infarcts can be achieved (94, 95). Sheep have similar AP profiles to man, including transmural dispersion of repolarization, but a transmural Purkinje system is present, different than man (93, 96–98). There are well-established models of acute ischemia and MI, as well as chronic MI with ventricular remodeling and HF (39–41). Importantly, these models do develop spontaneous VT/VF (48, 99).

#### Goat

The goat as an AF model is well-established. This model is produced by burst pacing of the atria, and is therefore analogous to the clinical correlate of paroxysmal atrial fibrillation, where the mechanism of AF is related to triggered ectopy originating from the pulmonary veins (29). This model has no underlying structural changes in the atria during short term pacing, but its electrical remodeling is well-defined. However, some structural remodeling (myolysis without fibrosis) occurs with longer term pacing, as does sustained AF (100). It has also been used to evaluate the effectiveness and proarrhythmic effects of antiarrhythmic drugs (101). Given its relatively uniform coronary anatomy between subjects, goat can be used in studies of MI (102).

### What Are the Specific Arrhythmogenic Conditions Studied Using Translational Large Animal Models?

#### Ischemia/Reperfusion and MI

Although canine models were developed first (103), and have provided significant mechanistic information including the paradigm of Phase 1 and 2 arrhythmias (104), ischemic models of cardiac arrest and MI in pig are now the more preferred model platform given anatomic similarities to man regarding coronary anatomy. As cellular studies require simulated ischemia, isolated heart and large animal models have not only historical significance in providing the earliest mechanistic data for studies directly looking at coronary occlusion and MI, but with the exceptions of some advantages of isolated whole heart preparations, are the only models which can faithfully model the complexity of hypoxia, acidosis, and autonomic changes associated with development of ischemia-induced VF (5, 105). Models of established MI without HF and acute ischemia in ischemic cardiomyopathy are created using various methods of coronary artery occlusion in pig, dog, and sheep (Table 1). Coronary occlusion by open ligation, intracoronary microembolism, or reversible balloon occlusion are all options depending on the question being asked.

#### Cardiopulmonary Resuscitation

Resuscitation from cardiac arrest is complex, with hemodynamic, autonomic, and electrophysiologic factors all playing a role. Furthermore, there are multiple etiologies of cardiac arrest, which can be difficult to initially determine clinically, impeding initiation of specific therapeutic interventions. Even when acute ischemia is the most likely cause, a mix of focal ischemia and global ischemia may promote different and varying arrhythmia substrates not accounted for by advanced cardiac life support (ACLS). The complexity of resuscitation from cardiac arrest in man can best be modeled in large animal models, where realistic resuscitation protocols can be reproduced. For example, the patient undergoing resuscitation from ischemia-induced VF initially undergoes regional ischemia (myocardial infarction), then global ischemia during VF, global reperfusion during resuscitation, and subsequent reperfusion of the culprit lesion. These phases have distinct electrophysiological properties that are not accounted for by current ACLS protocols, and the interplay of arrhythmia substrates and susceptibilities during each phase is unknown. All these phases can be incorporated into a large animal porcine model (22). *In vivo* porcine models have provided important results on resuscitation approaches and outcomes, although mechanistic information on arrhythmia formation and maintenance is less well-understood (49, 50, 53, 54). We recently reported that in a porcine translational model of ischemia-induced SCA that arrhythmia substrates were in fact dynamic during different phases of resuscitation that they were modified by therapeutic hypothermia. In addition, cardiac alternans was an important mechanism of recurrent VF during resuscitation (22). Regarding species specific models of cardiopulmonary resuscitation, the advantages of transmural and regional ion currents, more human like conduction system and lower susceptibility to VF in dog vs. pig is a consideration when evaluating outcomes or therapies (11, 14). However, given additional factors, particularly similar coronary anatomy, ability to perform chest compressions during cardiopulmonary resuscitation, and cost and housing considerations, and importantly successful translation of interventions to man, the pig has emerged as the primary model (9, 51, 52, 106, 107).

#### Heart Failure

Multiple HF models in pig, dog, and sheep exist, each with specific advantages and disadvantages. Pacing-induced HF in dog is a well-established model of dilated cardiomyopathy. Its mechanical, ion channel and electrical remodeling has been thoroughly investigated (35). It is titratable and relatively easy to induce reproducible degrees of HF. However, despites its fidelity to many aspects of clinical HF in man, its ready reversibility and mechanism of induction using tachypacing is relevant to a small population with tachycardia induced HF, making it potentially less attractive HF model (35, 108). This tachypacing model has also been used in pig and other large animals (30). Models of ischemic HF after MI from coronary occlusion or microembolism are well-established and validated (19, 32). Pig MI models are more reproducible, given their more uniform coronary anatomy and less prominent coronary collateral circulation vs. dog (11). As ischemic cardiomyopathy is highly clinically prevalent, these models have major translational importance. When microembolism techniques are used to induce ischemia, more uniform and controlled ischemic insults can be produced. This produces more controlled HF and is perhaps the preferred method in dog models of ischemic cardiomyopathy (19, 33).

#### Channelopathies

Much of our knowledge of regarding animal models of inherited channelopathies, such as the LQTS, comes from murine and small animal models. *Ex vivo* large animal models, typically recapitulating a channelopathy using pharmacology, have also contributed significantly to understanding mechanism (27). Acquired channelopathies can be related to disease (i.e., HF and hypertrophy), and/or drug induced. An important specific translational model of acquired long QT syndrome is the AV block dog which recapitulates acquired long QT syndrome in dogs which develop hypertrophy. This model is characterized by spontaneous TdP, with significant characterization of its ion channel remodeling, repolarization reserve and abnormal calcium cycling. It has been used as a preclinical test for drug induced TdP and to evaluate TdP mechanisms (23).

#### Other Arrhythmogenic Diseases

One example of specific model based on a genetic mutation is that of arrhythmogenic right ventricular cardiomyopathy in Boxer dogs. It is associated with a chromosome 17 variant, and the phenotype shares features consistent with human disease, including structural/pathological changes in the ventricle, significant electrophysiological remodeling, spontaneous ventricular arrhythmias, and SCD (25, 73). In addition, this model is characterized by VT of suspected right ventricle origin and structural right ventricular abnormalities. It is extremely well-characterized in terms pathology, structural, and electrophysiological remodeling, as well the arrhythmogenic phenotype.

#### AF

Importantly, there are multiple AF models, many providing important complimentary mechanistic insight into human AF, which have formed the basis for preclinical development of multiple pharmacologic and other surgical/ablation interventions (28, 29) (see Table 1). Models have been developed in dog, goat, pig and sheep, incorporating pacing-induced remodeling, HF, sterile pericarditis, mitral valve disease, and autonomic modulation, amongst others. Most have been very well-characterized by multiple groups, including underlying structural and electrical remodeling and determination of arrhythmia substrates and triggers. One of the most influential is Allessie's model of AF in the goat, where rapid atrial pacing promoted atrial electrical remodeling and eventually sustained AF (65). There are multiple AF models incorporating different aspects of human disease. These include burst rapid pacing of atria (as in paroxysmal AF) with or without ventricular pacing to produce combined AF with HF and atrial structural remodeling (a common but distinct clinical scenario) and a sterile pericarditis model (which mimics post-operative atrial flutter). This is produced by using talc to irritate the pericardium after mediansternomotomy. All these models have given insight into the multifaceted mechanisms in AF using different, complimentary approaches (28, 29).

In summary, several large animal species have been used to study disease states. Although pigs, dogs, goats, and sheep possess many similarities to cellular and whole heart electrophysiology to man, there are clear differences between species and the right model will always be a compromise (Figure 1). Well-established models of common arrhythmogenic diseases have been developed in these species, with each species having advantages and disadvantages for modeling these diseases (see Table 1 for details). Studies evaluating treatments and interventions for arrhythmogenic disease need to take into account the relative and sometimes complex advantages of each species and disease model.

## Lessons Learned: Successes and Pitfalls in Translation

It should be clear when discussing successes vs. failures in electrophysiological research that the majority of translational animal studies do not result in positive clinical trials as 60–80% of the translational studies which are ultimately tested in clinical trials fail (109, 110). It is also important to acknowledge that much large animal translational work suffers from methodological pitfalls that are well-recognized and common to all animal studies and certainly inherent in studies of arrhythmia mechanism. Sample size calculations and power analyses are infrequently reported or adhered to in translational studies, making it difficult to precisely estimate effect of studied interventions (110). Standard approaches used in clinical trials, like blinding and randomization, are inconsistently done, and those studies which do not report blinding or randomization strategies are more likely to be positive (110, 111). Taken together, this would be expected to overestimate positive results provided by subpar methodology, and along with publication bias, more likely to form an inaccurate basis for a clinical trial (110, 112). As young, healthy animals are often used in translational studies, this also makes the extension of results problematic, however, in many of the models discussed above regarding arrhythmia inducibility in disease, this is somewhat mitigated by replicating arrhythmia substrates in chronic animal models.

### Challenges in Large Animal Models

By their nature, large animal studies tend to be less mechanistic than those in *ex vivo* or smaller animal studies (such as transgenic mice). Given the complexity of large animal models, validating specific mechanisms represents a significant challenge and sometimes falls short, or more often is not attempted at all (110). Even when significant efforts are made to create a translational model with high fidelity to human disease and to fully characterize it, challenges remain to directly relate documented cellular mechanisms to whole heart arrhythmia mechanisms given the complexity inherent in any large animal model. *In vivo* large animal studies are costly, labor-intensive and ethically problematic (6, 113). Anesthesia considerations are particularly important as they can alter sympathetic modulation of arrhythmias, arrhythmia inducibility, ischemic insults, and anesthesia itself can be proarrhythmic (20, 114, 115). Cost is certainly a consideration when performing large animal translational experiments, particularly if purpose bread or genetically modified animals are being studied (69). Societal acceptance for using large animal models can be highly variable, but is generally low, and strict regulatory concerns must be addressed. Unfortunately, cost and ethical considerations can make it difficult to justify and perform large studies in these species, and many large animal studies suffer low power and resulting inaccurate point estimates of the outcome variables (110–112). All these considerations of design and implementation and potential compromises made must be considered when interpreting and applying results of large animal models to clinical trials.

### Challenges Specific to Determining Arrhythmia and SCD Mechanism and Testing Therapies in Translational Models

An important feature of all translational model platforms is that they either faithfully reproduce the relevant arrhythmia mechanisms in the disease in question if testing therapy, or are designed to determine new mechanisms in a disease. However, there are important considerations when evaluating the effect of an antiarrhythmic therapy in a large animal model. How well does the model link the antiarrhythmic effect observed to an established arrhythmia mechanism in the disease of interest? How well does the study characterize the arrhythmias and what is their mechanistic basis in the model? Are mechanisms being established or tested in *vivo* assessments or *ex vivo* using tissue/cells (32, 116). This can be particularly challenging when mechanisms can by dynamic and time dependent (such as in ischemia/reperfusion or autonomic modulation of arrhythmias), there are multiple mechanisms at play (as in HF, for example), and these mechanisms may be interdependent (117). Modeling both arrhythmia triggers as well as substrates are central to understanding development of reentrant excitation in man, as both are typically required. Models in which dispersion of repolarization can be quantified and directly related to development of conduction block have provided significant information, but mapping these arrhythmias can be challenging (118). A necessary trade off when testing therapies is that determination of specific electrophysiological substrates and the mapping of arrhythmia initiation oftentimes requires extensive invasive monitoring, and although may provide mechanistic information, this can be at the expense of compromising the model. For example, large animal models of cardiac alternans, an important mechanism of reentrant arrhythmia in ischemic heart disease and HF, have been used to evaluate efficacy of antiarrhythmic drugs to suppress alternans and arrhythmias (119, 120). These studies have provided valuable information on potential therapeutics, but not necessarily direct mechanistic insights. Triggered activity is an additional important arrhythmia mechanism which has been reproduced in translational models. However, directly relating triggered activity to its underlying mechanisms (calcium dysregulation in HF or altered repolarization) is more difficult in the whole heart than in simpler model platforms (121), sometimes limiting mechanistic insight when testing potential therapies. Additional conical arrhythmia mechanisms, such as increased automaticity, are also important to consider, but difficult to study *in vivo*. Finally, neural modulation of arrhythmia substrates, triggers, and automaticity is an important consideration and can be directly examined in experimental models (20, 78).

A particular problem with developing models to determine mechanisms and test therapies is that the occurrence of spontaneous arrhythmia in man *is spontaneous*. This is difficult to replicate in animal models. Although we know much about mechanisms of arrhythmia triggers and initiation, the “holy grail” is to be determined: why any individual would have a particular event at a particular time? Modeling of spontaneous arrhythmias and their suppression is highly important but rarely achieved as it requires specific monitoring, which is not a trivial task. Moreover, while arrhythmia inducibility using programmed electrical stimulation protocols or drugs is oftentimes used, its translational relevance is questionable. Many models utilized different protocols to induce arrhythmias, but it is critically important to understand the mechanism of the arrhythmia being studied as well as the protocol used to induce the arrhythmia to insure that findings match. Using standard programmed stimulation protocols during bradycardia to induce torsades des pointes (TdP) in models of LQTS (27) provides insight into mechanism, but performing rapid pacing to induce VT in that same model would not be as insightful. Rapid pacing protocols alone, without attempts to determine the etiology of the arrhythmia induced (alternans, triggered activity, for example), are routinely performed but lack mechanistic information.

In summary, large animal models of arrhythmia help us understand the relevance of previously defined *in vitro* arrhythmia mechanisms within the whole animal. However, performing translational studies in large animal models can be time and resource intensive while becoming less mechanistic in the process. Models of specific arrhythmia disease states have been developed, but reproducing human arrhythmia substrates in these models has been challenging. Spontaneous arrhythmias in these models are not uniform, and protocols to stimulate arrhythmias may be less translational. However, large animals models remain an important translational link between individual arrhythmia mechanisms and arrhythmia formation and management in the whole organism.

### Success Stories in Translation

#### Cardiac Arrest and Resuscitation

As cardiac arrest induces global multi-organ dysfunction, large animal models are particularly well-suited to study the complex interactions between ischemia-reperfusion in multiple organ systems and develop evidence based therapies. The first large animal model of cardiac resuscitation was in 1957, which described closed chest compression (122). The pig has emerged as the best model of resuscitation due to its physiologic parameters being similar to human, the ease of instrumentation, the ability for post-resuscitation neurologic testing, and the ability to perform CPR (51, 52, 55). Although the pig model suffers from all the challenges of large animal models described above, it is a model success due to its ability to provide mechanistic knowledge of resuscitation and its ability to test interventions in a challenging clinical setting. Many lessons learned from animal models have been incorporated into cardiopulmonary resuscitation protocols, tested in humans, and incorporated into guidelines, improving outcomes (123). Optimal delivery of CPR, including chest compression ratios and avoiding pauses in compressions, as well as compression only CPR where initially tested in pig models (51, 52). Years of work in cerebral resuscitation and protection by therapeutic hypothermia was tested in multiple animal models, until eventually being brought to the clinic (124).

#### Determining Mechanism of Arrhythmias in Ischemia/Reperfusion

Years of work in multiple translational models have firmly established basic mechanisms of arrhythmias in ischemia/reperfusion. Initial development of the paradigm of phases of arrhythmias was first determined in dogs, and this paradigm was reproduced in other models, notably a pig model of ischemia, showing different phases of ischemia and arrhythmia, consistent with human arrhythmia development during ischemia (42, 43). Particular underlying mechanisms of the arrhythmias observed were then further determined using other model platforms. For example, using the established paradigm, the etiology of decreased cell to cell coupling, conduction slowing and reentry arrhythmias demonstrated in type 1b arrhythmias in pig was later demonstrated to be in part attributable to connexin 43 remodeling in rat model of ischemia, with changes in Cx43 localization, expression and phosphorylation (125). This then formed the basis for additional large animal translational studies on gap junction modulation to suppress ventricular arrhythmia (42, 125). This is only one example of defining a mechanisms of ischemia/reperfusion arrhythmias using large animal models, amongst a wealth of others (5, 43).

#### Determining Mechanisms and Therapies of Arrhythmias in AF

Our basic understanding of mechanisms of AF initiation, maintenance, and variability in substrates and triggers in different diseases has also been firmly established using translational large animal models. Although not uniformly demonstrating spontaneous AF, multiple pacing-induced AF models in dog, goat, pig, initially based on Allessie's model of rapid pacing of atria in the goat, have provided significant insight into mechanism later allowing testing of therapies (65). The rapid atrial pacing model adapted to HF in AF model and a sterile pericarditis model, mimicking post-operative AF, amongst others, all have given insight into the multifaceted mechanisms in AF using different, complimentary models (64). These complimentary models were adapted to investigate different features of human disease, such as lone AF, paroxysmal, or AF in HF with structural remodeling. Differences in these models regarding remodeling of repolarization, heterogeneities of conduction, and structural remodeling, including fibrosis, have demonstrated the importance of different substrates in different clinical subtypes of AF and identified common themes providing insight into mechanisms and therapies for AF in man (28, 65). The careful characterization of arrhythmia mechanism in these models allowed development and preclinical evaluation of therapy based on established mechanisms (29, 126).

#### Establishing Treatment in HF With Mechanical Dyssynchrony

Translational studies investigating mechanisms underlying the clinical observation of worsening mechanical function in patients undergoing ventricular pacing and left bundle branch block were central to the development of cardiac resynchronization therapy for HF patients (127). Studies in large animal models evaluated both adverse and favorable electrical and mechanical remodeling using pacing strategies in heart failure (77) were subsequently tested in man, and bi-ventricular pacing is now standard therapy for subgroups of HF patients with low ejection fraction and conduction delay (128). A large body of evidence looking at effects of asynchronous ventricular activation on mechanical strain, causing adverse mechanical and electrical remodeling, also suggested potential for proarrhythmia using epicardial pacing (15, 129, 130). However, it is more likely that effects of reverse mechanical and electrical remodeling, improvement of HF and mitigation of ischemia is ultimately antiarrhythmic, and clearly improves HF related morbidity and mortality. Clinical studies show that HF patients treated with CRT-ICDs have less arrhythmia events and ICD shocks (131, 132).

### Challenges in Translation

When examining large animal translational studies which ultimately did not lead to clinical therapies or treatment, multiple themes arise. As with current clinical therapies for arrhythmias (ablation, antiarrhythmic drugs), translating interventions developed in large animals to long-term efficacy is highly challenging and must be considered. If an intervention is expected to induce reverse remodeling (as in biventricular pacing in HF), it can be expected to potentially mitigate long term arrhythmia substrates and triggers, but a one-time intervention (as with gene transfer interventions for specific reentrant arrhythmias) may only provide more short term improvements, particularly if underlying arrhythmia substrates continue to progress (133). Specific themes and examples of difficulties with translating findings in large animal models of arrhythmia and SCD are reviewed below:

#### Heterogeneities of Human Disease Not Adequately Modeled

Heterogeneities in human disease are not typically manifest in any specific animal models, and mechanisms identified or therapies tried can't be extended to all cases. HF is the clearest example where there are multiple different clinical phenotypes (ex. diastolic, systolic, HF with preserved ejection fraction, mixed) and etiologies (ex. ischemic, hypertensive, toxin-mediated, and other dilated cardiomyopathies), and each etiology and subtype is itself extremely complex. Addressing complexity with different, complimentary models, as has been done in AF is a clear example of developing mechanistic understanding by the different models. However, although there are models reflecting the heterogeneities in human disease, it is of course unrealistic to expect a specific therapy can be necessarily used in all patients, and when testing, very difficult to perform a trial with only specific subtypes of a disease are being identified and included, particularly with funding often through industry who may stress more global applications. So although large animal models have clearly informed our mechanistic understanding of the multifaceted mechanisms of arrhythmias in human HF, it remains difficult to extend testing of translation therapies in any one model to a heterogeneous population.

#### Translating Human Standard of Care to Animal Models

In clinical trials, patient care is optimized. For example, in HF studies patients receive close monitoring and better HF care than they might otherwise, so it is difficult to detect differences in an intervention. Gene therapy for improvement of left ventricular function and arrhythmias using SERCA2A overexpression has been tested in both small animal and larger translational models of HF and ischemia/reperfusion and has robust arrhythmic effects, based on well-defined mechanisms, and importantly did not promote proarrhythmia due to triggered activity (79, 134, 135). However, when in a careful well-executed clinical trial designed to determine effect of SERCA2a overexpression on heart failure, essentially the effect on HF was neutral, while arrhythmia susceptibility was not an outcome measure (136). In this trial, patients were well-matched with multiple underlying etiologies of HF and received state of the art medical care. Although absolutely necessary when designing a clinical trial, demonstrating efficacy in a population which is optimally managed requires a significant treatment effect, also more difficult to determine in a heterogeneous treatment population. Similar difficulties in establishing efficacy were observed in a clinical trial testing a novel peptide based therapy to maintain gap junction coupling to limit ischemia/reperfusion injury in ST elevation MI (80). A strategy of maintaining gap junction coupling during ischemia and reperfusion to suppress arrhythmias was tested in multiple large animal models, where both clear signals of limitation of infarct size and suppression of ventricular arrhythmias was shown (137–139). However, no improvement in myocardial preservation was observed in this carefully performed clinical trial. It was performed in a system where remarkably quick coronary reperfusion was achieved (door to balloon time approximately 30 min and symptom onset to first medical contact less than an hour), limiting ischemia time and exposure to treatment prior to reperfusion (80). With such exemplary clinical care, it would be very difficult to detect differences in myocardial preservation, as the majority of patients would not be expected to have significant long term myocardial damage.

#### Incompletely Elucidating Mechanisms

It is important to understand the arrhythmia mechanisms being studied by an intervention to insure its relevance when translating to human trials. Much of what we know about mechanisms of antiarrhythmic medications for resuscitation was tested and developed in other settings. There is ample preclinical data on mechanisms of lidocaine and amiodarone, but not during resuscitation. This may be in part why their utility was initially demonstrated and drugs where incorporated into wide clinical use, only later to be called into question, except in particular subgroups (140). A potential criticism of the strategy of pharmacologically maintaining gap junction coupling to prevent arrhythmias in ischemia/reperfusion in man, discussed above, was that despite the strong treatment effect demonstrated in translational animal models, the mechanism of action of the peptide tested remains incompletely understood. This is despite some mechanistic studies and significant biologically plausibility of its antiarrhythmic action and conflicting, but largely positive data regarding effects on myocardial preservation by maintaining gap junction coupling (141). In contrast, although also not completely elucidated, multiple translational studies supporting biventricular pacing were used to derive the mechanisms underlying mechanical and electrical reverse remodeling, supporting its clinical implementation (130, 142).

#### Translating Outcome Variables From Animal to Human

Oftentimes in performing translational studies, multiple outcome variables are examined to maximize knowledge gained in very time consuming, expensive work. However, when translating to clinical trials, only the most impactful or marketable outcomes are pursued. It is important to align primary outcomes from translational large animal studies to human trials. This is particularly important when outcomes evaluated in the animal trials may have been secondary outcomes, and the preclinical study which may not have been designed to specifically demonstrate differences between a variable of interest that was later was tested in a clinical trial. Sometimes there is a mismatch between the disease or specific target tested in animal models and primary outcome to which it is applied in a clinical trial (wrong target, wrong disease). This is oftentimes due to necessary compromises made in clinical studies, or that a target may not be able to be practically pursued, and if benefit in more than one outcome has been demonstrated, choices need to be made. Spinal cord stimulation has been used for years to treat chronic pain and as a therapy decrease refractory angina in man. A wealth of data demonstrated the efficacy of spinal cord stimulation in translational models to suppresses ischemia induced ventricular arrhythmias where the initial antiarrhythmic signal tested during acute ischemia with and without establishment of HF (18, 143). Improvement in indexes of ventricular remodeling was an outcome in these preclinical studies. These findings were robust and reproduced in multiple studies and models, providing strong evidence of both the antiarrhythmic and reverse ventricular remodeling efficacy of spinal cord stimulation and had significant mechanistic basis. Primary and secondary outcomes of the clinical trial were related to LV function, and although arrhythmias were examined in the majority of the patients and were not different between treated and untreated patients, the design of the study was not necessarily to asses those outcomes. Similarly, in preclinical studies of peptide strategies to maintaining gap junction coupling during ischemia and reperfusion a strong signal for antiarrhythmic effects was demonstrated in multiple large animals, from multiple groups of ischemia reperfusion, which was not the primary outcome when tested in a clinical trial (80).

#### Translation of the Intervention

Because of practical concerns of cost, judicious use of animal resources, and time constraints, large animal models testing therapies are optimized to demonstrate effectiveness. However, this occasionally results in interventions that can't be delivered as well-clinically and additionally, compliance of patients with tested therapies is a challenge. An example is use of spinal cord stimulation in man, which has significant translational promise as an antiarrhythmic and HF therapy, but its clinical implementation is not trivial. This is despite extensive preclinical dosing development in translational experiments (144). Investigators could not deliver the same duration or type of stimulation as was done in animals, and subjects couldn't be adequately blinded as patients could often tell and were bothered by paresthesias when the stimulator was on. Enrollment was stopped due to futility (145). Of note, a non-controlled study using a different stimulation protocol did show a signal of benefit, suggesting the negative results may have in part been explained by the stimulation protocol used (146). Another example of difficulty in translating therapy in man is gene delivery, where there has been exciting data in translational models demonstrating great therapeutic potential, but has proven difficult to implement successful in clinical trials. In man, gene delivery is challenging and expensive, particularly if not focused to a specific anatomical region. The large doses needed is a limitation. More targeted delivery to a scar and MI border zone or atrial epicardium may be more practical and likely to succeed in the near future (147). Resuscitation studies have also suffered from difficulty in delivery of interventions. Drug delivery occurs quicker in animal models of cardiac arrest, which likely improve outcomes compared to clinical studies (148). Furthermore, animal models were used in device testing for mechanical CPR, leading to a large clinical trial of prehospital mechanical CPR which did not show an improvement of survival (149, 150).

In summary, large animal models of arrhythmia have been both successful and challenging to translate into medical practice. Large animal studies often suffer from decreased scientific rigor of their human counterparts and focus on outcomes that might not be as pertinent in human studies. However, large animal models have been highly influential, with characterization of arrhythmia mechanisms, establishing novel therapies, and translation of diseases with low survival being the most successful. Common challenges of large animal studies tend to revolve around common themes: outcome variables not directly translatable to humans, incompletely elucidated mechanisms, and clinical standard of care. Some of these pitfalls may be avoided with careful study design, and understanding of outcomes that would likely be tested in human studies. A summary of Translational successes and pitfalls are reviewed in Table 2.

**Table 2:** Translational successes and pitfalls.

**Translation advantage**	**Implications for human study**	**Examples (see text for discussion)**
**TRANSLATIONAL APPROACH SUCCESS**		
Choosing a model that faithfully represents human disease and arrhythmia mechanisms	Using right platform for right disease optimizes implementation of treatment. Typically requires careful characterization of a disease model and not utilizing otherwise normal animal subjects	Coronary artery occlusion induced cardiac arrest in pig (21)
Maximizing the experimental design including performing power analyses, blinding and randomization that may not be possible in clinical trials	Allows more precise understanding of the effect size of the treatment to better design human trials Rigor in animal work more likely to produce externally valid results when making decisions whether to proceed with testing in man Limit overestimation of findings and reporting of only positive results	Porcine Resuscitation Models to optimize CPR delivery (51)
Developing a model that faithfully reflects the heterogeneities of human disease and reproduce arrhythmia phenotypes of interest	Allows specific targeting of mechanisms and interventions to subpopulations with a particular disease or subtype	Complimentary AF models in dog: Sterile pericarditis, combined HF and pacing-induced AF, and atrial pacing induced AF (28)
Ability to reproduce mechanistic models over multiple studies reproduced by multiple investigators	Reproducibility of data insures more likely to be successfully translated to human disease and internal and external validity of findings	Canine pacing induced mechanical and electrical remodeling (77)
Development of a study protocol makes every effort to faithfully reproduce clinical intervention	Anticipating planned implementation in man optimizes translational potential of the study	Porcine resuscitation models to optimize CPR delivery (51)
**Translation challenge**	**Implications for human study**	**Example**
**TRANSLATIONAL APPROACH PITFALLS**		
Difficulty in applying treatment strategies developed in models to human treatment	Therapies being tested in animal models typically have optimized timing and delivery which are then difficult to fully implement, reproduce and test in man	Dog models of spinal cord stimulation to suppress arrhythmia and improve mechanical function in HF (78) Gene therapies to suppress ventricular arrhythmias in HF (79)
Primary experimental endpoints in models not necessarily same ones pursued in clinical trials	Likelihood of failure if not directly testing primary outcomes of animal studies in man, but rather other signals of benefit observed	Strategies of gap junction preservation in acute MI (80)
Mechanistic basis for therapy not firmly established and are assumed based on incomplete data	Risk that both positive and particularly negative trials will provide little information for further development of the therapy and misinterpretation of the clinical effect	Strategies of gap junction preservation in acute MI (80)
Optimized standard therapies in clinical trials make showing improvements achieved in models difficult to reproduce	Difficult to show benefit, even if one exists. Requires that before basing trials on animal studies, they have also modeled optimal therapies in those studies	Studies in human HF, including autonomic modulation and gene therapies
Heterogeneities in human disease not adequately modeled	If targeting a a specific mechanism or phenotype, it may be difficult to show benefit in the overall population, and may exclude a potentially promising intervention for subgroups of patients	Studies of disease with multiple phenotypes and variable severity, such as HF and AF

*Successful translational approaches and pitfalls to be considered when designing large animal translational studies and/or basing data derived from them into clinical trial design*.

## Recent Advancements

Although large animal models are oftentimes limited mechanistically due to inherent limitations in invasive arrhythmia mapping and establishing direct mechanistic links in a highly complex system, there have been significant strides made recently to improve methodology and provide more mechanistic insight.

### Genetic Modification in Large Animal Models of Arrhythmia

A porcine model of Brugada syndrome was produced by Park et al. in which they engineered an early truncation mutation in SCN5a (E555X) to produce SCN5a haploinsufficiency (70). Although this model did not have *in vivo* arrhythmias, in Langendorf experiments, temperature-dependent changes in conduction and arrhythmias induced by rapid pacing were demonstrated and consistent with Brugada syndrome. A criticism of the model was that the pigs did not exhibit classic changes observed in the Brugada ECG. Due to cost and housing limitations, investigators were unable to allow the pigs to mature into adulthood so perhaps the phenotype was not fully realized, and pigs minimally express the transient outward potassium current, which has been demonstrated to be mechanistically related to arrhythmia susceptibility in Brugada patients (69). There are also multiple mutations which create a Brugada phenotype. However, this model represents a major advance in studying Brugada syndrome. Given that multiple transgenic pig models have been produced, it is likely it will be used more as a model platform for additional arrhythmogenic disease in the future (9, 151). Transgenic goat model of cardiac- specific overexpression of TGFB1 with atrial fibrosis and increased susceptibility to atrial fibrillation has also been developed (152).

Genetically modified large animals serving as model platforms to understand mechanism or test therapies have clear advantages over rodent models, where although signaling pathways and underlying mechanisms can be better delineated, its relevance to the human electrophysiological phenotype is oftentimes questionable. However, establishing large animal models are expensive and difficult to perform. By their nature, they are targeting a specific disease or mutation, so not necessarily relevant to the more common complex arrhythmogenic diseases, such as HF (151). Importantly, the phenotypes produced do not tend to fully represent the human disease, which is not necessarily surprising given that even in arrhythmogenic disease thought to be monogenetic, and most potentially amenable to be reproduced in large animal models, multiple modifying factor (genetic as well as environmental) are at play, complicating phenotypical penetrance in man. This would presumably be only more problematic in large animal models (153).

### Targeted Gene Delivery

Gene transfer technologies have been applied to translational large animal models for some time and continue to provide significant mechanistic insight into arrhythmias and have potential as therapies. Multiple targets have been tested in large animal models, primarily targeting reentrant mechanism by disrupting reentrant circuits locally by prolonging repolarization (154) or enhancing conduction in both ventricle and atria (44, 58) and have generally found to be antiarrhythmic. An additional strategy is normalizing calcium cycling using gene overexpression of SERCA2a during ischemia/reperfusion (135) or HF, in which the antiarrhythmic mechanisms of normalizing calcium cycling have been demonstrated in smaller animals (134), but yet to be fully elucidated large animal translational models. In a pig model of complete heart block, a “biological pacemaker” was created using an adenoviral vector delivery of the of transcription factor T-box 18 to convert ventricular myocytes into pacemaker cells. The investigators demonstrated improved heart rate response to exercise, and although the duration of effect was only on the order of 2 weeks, they proposed this could represent a temporizing therapy for patient with lead infections requiring explanation, antibiotics and time prior to reimplantation (155). Although targeted gene delivery has potentially significant therapeutic potential, this has yet to be realized, and has significant limitations which need to be overcome (133).

### *In vivo* Optical Mapping

A particularly exciting recent advancement is the potential use of optical mapping *in vivo* in large animal models. Optical mapping utilizes voltage sensitive fluorescent dyes to measure cardiac action potentials with high spatial and temporal resolution (156). It has provided significant insight into mechanisms of arrhythmias in both normal and disease models. It has traditionally required *ex vivo* preparations due to limitations of using voltage sensitive dyes in blood perfused preparations and requirements for mechanical or pharmacological motion control of the preparations; however, *in vivo* optical mapping in small animals, also incorporating calcium indicators has been done (157) and was initially reported in canine using a fiber optic probe and intracoronary injection of dye. This allowed action potentials to be recorded from a 4.5 vs. 4.5 cm area of the LV epicardial surface and study *in situ* mechanisms of VF and response to defibrillation (158). Recently, a new method for *in vivo* optical mapping of pigs has been reported (159). Using two near-infrared voltage sensitive dyes, the authors were able to demonstrate that, *in vivo*, ratiometric signals could reduce motion artifacts, allowing for mapping of activation (at faster CLs) and VF dynamics. The addition of a fiberoptic system allowed measurements of repolarization and action potential maps with high fidelity from a more limited area of the heart. Clearly, this can become a very valuable tool for studying arrhythmia mechanisms in large translational models, and it is highly likely that further refinement of this system, such as incorporating multidetector panoramic or intrachamber imaging could allow for even greater mechanistic evaluations (160).

## Conclusions

Large animal models of arrhythmia have been instrumental to the translation of specific *ex vivo* arrhythmia mechanisms into whole organism systems to both determine relevance of indentified arrhythmia mechanisms and approaches to their treatment in humans. Large animal models have led to changes in established therapy, novel approaches to treatment, and continue to be a necessary vessel for introduction and testing of novel techniques and therapies. Several large animal species are available with multiple specific disease models. Understanding the pros and cons of these different species and disease models is paramount when interpreting data derived from translational models and before testing novel therapies in man. By understanding past successes and challenges, large animal models of arrhythmias can be further developed and refined to better translate novel ideas into therapy. Studies in large animal models of arrhythmia will continue to be an essential tool to further our understanding of arrhythmias and design therapies to improve outcomes in patients suffering from cardiac arrhythmias.

## Author Contributions

Both authors contributed equally to the conception, design, and writing of the review.

### Conflict of Interest Statement

JP received grant from ZOLL Foundation. JP and LW received a gift of investigational compounds from Zealand Pharma.
